# Atrial Fibrillation and Cancer: Pathophysiological Mechanism and Clinical Implications

**DOI:** 10.3390/jcm14155600

**Published:** 2025-08-07

**Authors:** Alfredo Mauriello, Adriana Correra, Vincenzo Quagliariello, Martina Iovine, Pierpaolo Di Micco, Egidio Imbalzano, Francesco Giallauria, Antonio Giordano, Vincenzo Russo, Antonello D’Andrea, Nicola Maurea

**Affiliations:** 1 Division of Cardiology, Istituto Nazionale Tumori—IRCCS—Fondazione G. Pascale, 80131 Napoli, Italy; quagliariello.enzo@gmail.com (V.Q.); martina.iovine@istitutotumori.na.it (M.I.); n.maurea@istitutotumori.na.it (N.M.); 2 Intensive Cardiac Care Unit, “San Giuseppe Moscati” Hospital, ASL Caserta, 81031 Aversa, Italy; adrianacorrera@gmail.com; 3 AFO Medicina, UOC Medicina Interna, P.O. Santa Maria delle Grazie, ASL NA2 Nord, 80078 Pozzuoli, Italy; pdimicco@libero.it; 4 Department of Clinical and Experimental Medicine, University of Messina, 98122 Messina, Italy; egidio.imbalzano@unime.it; 5 Department of Translational Medical Sciences, “Federico II” University of Naples, via S. Pansini 5, 80131 Naples, Italy; francesco.giallauria@unina.it; 6 Sbarro Institute for Cancer Research and Molecular Medicine, Center for Biotechnology, College of Science and Technology, Temple University, Philadelphia, PA 19122, USA; antonio.giordano@temple.edu; 7 Cardiology Unit, Department of Medical and Translational Sciences, University of Campania “Luigi Vanvitelli”, Monaldi Hospital, 80131 Naples, Italy; vincenzo.russo@unicampania.it; 8 Cardiology and Intensive Care Unit, Department of Cardiology, “Umberto I” Hospital, 84014 Nocera Inferiore, Italy

**Keywords:** atrial fibrillation, cancer, anticoagulant, thromboembolism, arrhythmias

## Abstract

Atrial fibrillation is the most frequent arrhythmia in elderly subjects. Cancer currently represents one of the most important causes of mortality and morbidity in the world. Often, the two pathologies coexist. Several pathophysiological mechanisms can lead to an increased risk of atrial fibrillation and cancer. Additionally, the same therapies used for cancer can increase the risk of developing atrial fibrillation. Our review aims to describe the pathophysiological mechanisms that promote the development of atrial fibrillation in cancer patients and explain the therapeutic opportunities and challenges of treating atrial fibrillation in patients with cancer.

## 1. Introduction

Atrial fibrillation (AF) is one of the most common sustained cardiac arrhythmias in adult patients characterized by high healthcare costs, morbidity, and mortality, due to its involutions. AF is a supraventricular arrhythmia characterized by loss of effective atrial contraction caused by uncoordinated high frequency electrical activity [1]. The electrocardiogram (ECG) highlights the absence of regular atrial contraction, with loss of P waves, and an irregular ventricular activation [2]. AF is an increasingly frequently diagnosed condition among cancer patients, resulting in a worsening of the prognosis of these patients. Currently, cancer represents the fourth cause of death from noncommunicable diseases worldwide and the sixth cause of death in general population, representing one of the most societal, public health, and economic problems in our time [3]. Cancer patients are at increased risk of developing AF, as these two conditions share risk factors and inflammation in their pathogenesis [4]. Moreover, several chemiotherapic drugs, with recognized cardiotoxicity activity, such as anthracyclines, and radiotherapy are known to be arrhythmogenic; however, the mechanisms remain unclear [4]. Further research is needed better to understand the pathophysiology of AF in cancer patients to establish prevention and treatment strategies specific to this population. Our review aims to describe the pathophysiological mechanisms that intertwine the two pathologies and explain the therapeutic opportunities and challenges of treating AF in patients with cancer.

## 2. Atrial Fibrillation and Cancer: Pathophysiological Mechanism

Several risk factors, such as age, arterial hypertension, diabetes mellitus, heart failure, coronary artery disease, and valvular heart disease are considered for developing AF [5,6]. Furthermore, AF is promoted by inflammatory mechanisms, which may represent common risk factors shared between AF and cancer [7]. An increased risk of AF has been observed in cancer patients [4]. In addition to the aforementioned risk factors, this heightened risk was previously thought to arise from either the medical and surgical treatments for cancer or from the cancer itself directly affecting cardiovascular tissue through compression or infiltration [7].

There is a bidirectional relationship between cancer and AF. Patients with recent-onset AF appear to be at increased risk of developing cancer in subsequent years [8], while patients with recent cancer diagnosis are more likely to develop AF. The highest incidence of AF is during the first 90 days of cancer diagnosis [9]. Even if a cancer is mild, the risk of AF does not end with diagnosis or treatment. This suggests that arrhythmia develops in cancer in complex ways [10].

AF develops in a complex manner, with several factors, including underlying conditions, initiating events, and influencing elements, all working together to cause and maintain it [11]. Atrial remodeling, such as electrical, structural, and contractile remodeling, are recognized as central to most acquired forms of AF [12].

AF and cancer are very common in elderly patients. Advanced age is a common risk factor for both conditions [13].

Therefore, a newly recognized chronic, low-grade systemic inflammation seems to be the cause of the age-related increase in cancer cases among the elderly and is linked to ‘inflammaging’ [13,14]. Authors propose the use of new biomarkers, such as DNA methylation, glycomics, metabolomics, and lipidomics that are capable of assessing biological versus chronological age in metabolic diseases.

The fundamental basis for numerous risk factors and pathogenetic mechanisms in both AF and cancer is, in the majority of instances, chronic inflammation. This aligns with the understanding that only a mere 10% of cancer cases are attributed to germline mutations; instead, most cancers arise from acquired factors, notably environmental cues, which are often intimately associated with chronic inflammatory states [7].

Persistent infections contribute to about 20% of cancers, with examples like Helicobacter pylori causing stomach cancer and hepatitis B/C viruses leading to liver cancer. While the immune system typically clears pathogens, cancer-causing ones often avoid this, leading to chronic infections that promote tumor-fueling inflammation [15].

Chronic inflammation caused by autoimmune disorders can raise cancer risk. For example, patients with inflammatory bowel disease are more prone to colorectal cancer because of the tumor-promoting effects of their ongoing gut inflammation [16].

Humans are exposed to many environmental factors that can cause persistent, though often mild, inflammation.

Among these are tobacco smoke [17], obesity and dyslipidemia [11,18], and alcohol intake [19].

Also, treatments aimed at killing cancer cells actually trigger inflammation that can help the tumor. When chemotherapy or radiation destroys many tumor cells, the dying cells release debris or molecules called DAMPs (damage-associated molecular patterns). These DAMPs then stimulate immune cells to produce pro-inflammatory cytokines, which are signaling molecules that, unfortunately, can support tumor growth, through angiogenesis, and the spread of metastasis [20,21].

Various pharmacologic cancer treatments have been implicated in the development of AF, such as anthracyclines, tyrosine kinase inhibitors, alkylating agents, anti-metabolites, human epidermal growth factor receptor 2 (HER-2) antagonists, cyclin-dependent kinase 4 and 6 (CDK4/6) inhibitors, BRAF/mitogen-activated extracellular signal-regulated kinase (MEK) inhibitors, immune checkpoint inhibitors, chimeric antigen receptor T-cell therapy (CAR-T) cell therapies and lenalidomide. Table 1 summarizes proposed mechanisms of several drug classes that contribute to the development of AF.

The connection between cancer and AF appears to center on the inflammasome. The innate immune system has complex structures within our cells that act as internal sensors for pathogens [32].

Of particular interest is the NLRP3 inflammasome, which has gained significant attention recently. This is due to its crucial role in inflammasome signaling and its involvement in various diseases, including AF [33]. Systemic inflammation and epicardial fat produce and secrete many pro-inflammatory cytokines (tumor necrosis factor-alpha, interleukin-6) and mediators (activin A and matrix metalloproteinases), which cause pro-inflammatory and pro-fibrotic state in the atrial myocardium [11]. Cardiac fibrosis also causes an alteration in the cytoarchitecture of the myocardium. There is a separation of the myocardial cells with an alteration in the normal conduction system that favors the development of ectopic foci that can determine the onset of arrhythmias. AF itself promotes structural remodeling creating a long-term vicious cycle that contributes to the development of persistent forms of AF [12].

**Figure 1:**
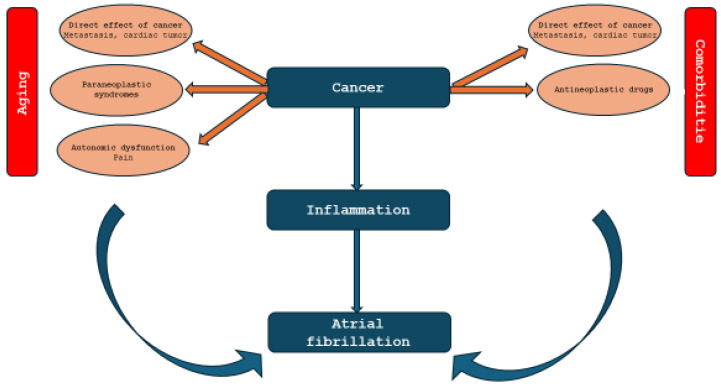
Relationship between cancer, inflammation, and atrial fibrillation. Blue: core pathological link; orange: cancer-related mechanisms contributing to atrial fibrillation; red: overarching background factors of atrial fibrillation.

Conditions like hypertension, obesity, diabetes mellitus, and gut dysbiosis can activate the NLRP3 inflammasome and other inflammatory signals, such as TNF-a and IL-6, within the heart’s atrial cells. NLPR3-mediated inflammation drives cancer initiation, immunosuppression, growth, and metastasis. This inflammation state leads to damage in the atria, perpetuating AF. Importantly, this NLRP3 inflammasome activation within atrial cardiomyocytes is a main mechanism of AF development. Furthermore, when the NLRP3 inflammasome is activated in immune cells that infiltrate the atria, such as macrophages, it also contributes to AF progression [34] (Figure 1).

## 3. Management of Atrial Fibrillation in Cancer Patients

The European Society of Cardiology (ESC) guidelines on AF do not clearly address the issue of the management of AF in patients with cancer [2]. Managing AF in cancer patients presents several significant challenges. These patients often face a high risk of bleeding, and there are numerous drug–drug interactions (DDIs) between antineoplastic agents and anticoagulants. Furthermore, there is a lack of validated scoring systems specifically for this patient subgroup and a dearth of randomized controlled clinical trials to provide robust scientific evidence [35]. Consequently, the optimal management of AF in cancer patients remains an ongoing challenge.

### 3.1. Thromboembolic Risk

The ESC guidelines on AF state that a patient experiencing a first episode of AF, if hemodynamically stable, should be treated with drugs for rate control, rhythm control, and prevention of cardioembolic stroke if their CHA_2_DS_2_-VASc (congestion, hypertension, age ≥ 75, diabetes mellitus, stroke, vascular disease, age between 65 and 74; female sex) score is ≥1 [2]. However, the CHA_2_DS_2_-VASc score is designed to identify low-risk patients for whom anticoagulant treatment should be avoided, and there is little evidence in the literature on its predictive value in patients with cancer [36].

D’Souza et al. [37] showed in a large Danish cohort of 122,053 patients with incident AF, of which 10% had a recent diagnosis of cancer, that bleeding risks were higher than stroke/systemic embolism risks in patients with recent cancer and a CHA_2_DS_2_-VASc score of 0, whereas both stroke/systemic embolism and bleeding risks were higher in patients with cancer and a CHA_2_DS_2_-VASc score of 1 compared with noncancer patients. The cumulative incidence of thromboembolism and bleeding at 2 years in patients with recent cancer compared with those without was higher and increased with increasing CHA2DS2-VASc score.

Leader et al. [38] in their retrospective cohort study divided patients into four subgroups: AF and cancer (n = 1411), AF and no cancer (n = 4233), no AF and cancer (n = 4233), and no AF and no cancer (n = 19,421). Systemic thromboembolism (STE) at 12 months was the primary endpoint, with a median follow-up of 3 years. The results demonstrated that the 12-month cumulative incidence of ATE was highest in the AF and cancer cohort (2.13%; 95% confidence interval (CI): 1.47–2.99) than in the AF and no cancer cohort (0.8%, 95% CI: 0.56–1.10) [(hazard ratio (HR) = 2.70 (95% CI: 1.65–4.41)]. One finding to highlight is that, regardless of anticoagulant therapy, the overall incidence of ATE was higher than the incidence of bleeding in these patients with newly diagnosed cancer. Therefore, venous thromboembolism (VTE) risk at 12 months was higher in the cancer patients when compared to AF and no AF cohorts without cancer. Patients without AF had a higher 36-month survival than the cancer population with AF.

Raoiseuras-Roubin et al. [39] in their registry, the CardioCHUVI-AF (Retrospective Observational Registry of Patients With Atrial Fibrillation From Vigo’s Health Area), confirmed these results. They conducted a retrospective observational Spanish study including 16,056 patients with AF (of which 1137 patients had AF and cancer) with a follow-up period of 4.9 years. In patients with AF and cancer not receiving anticoagulant therapy, the risk of STE was underestimated when the CHA2DS2-VASc score was used to assess the risk of thromboembolism. In contrast, patients with cancer and a CHA2DS2-VASc score = 1 had a risk similar to that of patients with a CHA2DS2-VASc score ≥ 2, and only patients with a CHA2DS2-VASc score = 0 had a very low risk of embolic events.

### 3.2. Bleeding Risk

The ESC guidelines on AF claim that bleeding risk scores should no longer be taken into account when setting up anticoagulant therapy [2]. However, it needs to be considered that the HAS-BLED (hypertension, abnormal liver and renal function, stroke, bleeding, labile international normalized ratio, elderly, drugs) score does not account for thrombocytopenia and the risk of intracerebral metastasis and should be used with caution in assessing the risk of bleeding in patients with cancer [40]. Therefore, the specific consensus for the subpopulation of patients with cancer is believed to consider still the scores for the evaluation of the bleeding risk [41]. A HAS-BLED score <3 and a platelet count >50,000/uL are considered safe [41].

### 3.3. Interactions Drugs

When talking about drug interactions, we tend to roughly distinguish between two main mechanical categories: pharmacodynamic interactions and pharmacokinetic interactions [42].

Treatment with vitamin K antagonists (VKAs) requires significant consideration of multiple food and DDIs. Despite fewer interactions with new oral anticoagulants (NOACs), it is necessary to consider the pharmacokinetic interactions of concomitantly administered drugs and comorbidities when prescribing NOACs [43,44].

Several NOACs present a meaningful interaction involving significant gastrointestinal re-secretion mediated by a P-glycoprotein (P-gp) transporter after absorption in the gut. P-gp is also involved in the active renal secretion of NOACs. Several molecules have a competitive inhibition role of the P-gp pathway that will result in increased plasma levels. It is essential to consider that many drugs used in AF patients are P-gp inhibitors (e.g., verapamil, dronedarone, amiodarone, ranolazine, and quinidine) [45]. Therefore, CYP3A4-type cytochrome P450-dependent elimination is relevantly involved in the hepatic clearance of rivaroxaban and apixaban [46]. It is usually not recommended to take NOACs with medications that strongly block both P-gp and/or CYP3A4, as this can increase NOAC levels. On the other hand, powerful activators of P-gp and/or CYP3A4 (such as rifampicin and carbamazepine) will drastically reduce NOAC concentrations in the blood. Therefore, using these strong inducers with NOACs must be avoided, or administered with extreme care and constant observation [43]. In conclusion, it is crucial to remember that many chemotherapeutic drugs are substrates of P-glycoprotein (P-gp) and various cytochromes [43]. Therefore, extreme caution is necessary when co-administering anticoagulants and chemotherapy regimens due to potential drug interactions [47].

### 3.4. Choice of Anticoagulant Regime

Over the years, several studies [48,49,50,51] have attempted to evaluate the efficacy and safety of an anticoagulant regimen in patients with AF.

Although low molecular weight heparin has been the anticoagulant of choice in the prevention of VTE for patients with cancer based on its superiority over warfarin, its use in thromboembolism in the context of AF/flutter is currently not supported by studies evaluating its efficacy in this patient setting [52].

The use of warfarin in patients with cancer and AF is not supported, as cancer patients have difficulty reaching the international normalized ratio target due to altered metabolism and numerous drug interactions between warfarin and chemotherapeutics [49].

Lee et al. [49], in their prospective study including 2168 consecutive nonvalvular AF patients with newly diagnosed malignancies, aimed to evaluate the composite endpoints, including major adverse cardiac events (MACEs) and major bleeding. During the first year after the cancer diagnosis, oral anticoagulant therapy did not improve the composite endpoint because of poor international normalized ratio control caused by cancer treatment (*p* = 0.181). However, after 1 year of diagnosis of cancer, there is a reduction in composite endpoint (*p* = 0.026).

Shah et al. [48], in their randomized clinical trial including 16,096 nonvalvular AF patients with cancer, aged mean 74 years, aimed to evaluate the effectiveness and safety of NOACs versus warfarin, as well as comparisons of NOACs. Bleeding rates were similar in rivaroxaban (HR 1.09 95% CI 0.79–1.39) and dabigatran (HR 0.96 95% CI 0.72–1.32) users compared with warfarin users, while those given apixaban had lower rates (HR 0.37 95% CI 0.17–0.79). Ischemic stroke rates did not differ among anticoagulant users. The rate of VTE was lower among rivaroxaban (HR 0.51 95% CI 0.41–0.63), dabigatran (HR 0.28 95% CI 0.21–0.38), and apixaban (HR 0.14 95% CI 0.07–0.32) users compared with warfarin users.

Deitelzweig et al. [50], in their retrospective observational study, including 40,271 patients, aimed to evaluate the risk of stroke/systemic embolism and major bleeding among AF patients with active cancer. Apixaban is safer for stroke/systemic embolism (HR: 0.59; 95% CI: 0.45–0.78) and major bleeding (HR: 0.58; 95% CI: 0.50–0.68) when compared with warfarin; dabigatran and rivaroxaban had similar risks of stroke/systemic embolism (dabigatran: HR: 0.88 [95% CI: 0.54–1.41]; rivaroxaban: HR: 0.82 [95% CI: 0.62–1.08]) and major bleeding (dabigatran: HR: 0.76 [95% CI: 0.57–1.01]; rivaroxaban: HR: 0.95 [95% CI: 0.85–1.06]). Comparing different NOACs revealed variations in stroke/systemic embolism and major bleeding risks. However, the treatment benefits across all NOACs comparisons held steady regardless of the specific cancer type.

Mariani et al. [51], in their meta-analysis, aimed to evaluate the efficacy and safety of NOACs vs. VKAs in cancer patients with AF. Nine studies were considered. A total of 46,424 NOACs users and 182,797 VKA users were included. The use of NAOCs was associated with a reduction risk of ATE or any stroke (relative risk (RR) 0.65; 95% CI 0.52–0.81; *p* = 0.001), ischemic stroke (RR 0.84; 95% CI 0.74–0.95; *p* = 0.007), and hemorrhagic stroke (RR 0.61; 95% CI 0.52–0.71; *p* = 0.00001), compared to the warfarin group. The use of NOACs reduced risks of major bleeding (RR 0.68; 95% CI 0.50–0.92; *p* = 0.01) and gastrointestinal and intracranial bleeding (RR 0.64; 95% CI 0.47–0.88; *p* = 0.006). Compared to warfarin users, NOACs determined a non-statistically significant risk reduction in the outcomes of major bleeding or non-major clinically relevant bleeding (RR 0.94; 95% CI 0.78–1.13; *p* = 0.50) and any bleeding (RR 0.91; 95% CI 0.78–1.06; *p* = 0.24).

However, the prescribers of NOACs must take into account the various variables that determine the reduction in the dosage, including age, body weight, and, especially, renal function. In patients with severely impaired renal function, with a filtration rate less than 15 mL/min, the use of apixaban, rivaroxaban, and edoxaban is contraindicated, while dabigatran is contraindicated with a filtration rate of less than 30 mL/min [53,54,55,56].

The coexistence of cancer increases both thromboembolic and major bleeding risks [57,58], so a multidisciplinary team discussion is needed to balance the equilibrium between thromboembolic (T) and bleeding (B) risks, interactions (I) among drugs, and patient (P) preferences included in the “TBIP” strategy.

## 4. Future Perspectives on Management of Atrial Fibrillation in Cancer Patients

### 4.1. Sodium–Glucose Cotransporter Two Inhibitors

Dapagliflozin, is a sodium–glucose cotransporter 2 (SGLT2) inhibitor initially developed for the treatment of type 2 diabetes mellitus. Only dapagliflozin, among other SGLT2 inhibitors, has emerged as a potential agent for mitigating chemotherapy-induced cardiotoxicity, including in non-diabetic populations [59]. Importantly, emerging preclinical and early clinical studies also suggest that dapagliflozin may mitigate chemotherapy-induced cardiotoxicity, an effect particularly relevant in cancer patients undergoing anthracycline or HER-2-targeted therapies [60].

Quagliariello et al. [61], in their preclinical study, observed that in female mice receiving doxorubicin followed by HER-2-blocking monoclonal antibody, the administration of dapagliflozin significantly saved the ejection fraction and reduced both radial and longitudinal strain impairment in mice treated with the doxorubicin–HER-2 inhibitor combination (*p* < 0.001). Therefore, levels of myocardial NLRP3, MyD88, C-X-C chemokine receptor type 4, Heart-type Fatty Acid-Binding Protein, interleukin-1β, and troponin-T were significantly lower in the dapagliflozin-treated group compared to the chemotherapy-only group.

### 4.2. Factor XI Inhibitors

Currently, there are only a few post hoc analyses on the role of dapagliflozin in preventing AF in the noncancer population [6]. Given the role of inflammatory factors in AF pathogenesis, preclinical and clinical studies evaluating the effects of dapagliflozin in preventing AF in cancer patients would be necessary.

Currently, there are several phase II and phase III studies underway regarding the use of factor XI inhibitors in cancer patients to prevent or treat VTE events, prevent catheter-related thrombosis and prevent arterial thromboembolic events, summarized in Table 2.

The potential long-term benefit of factor XI inhibition in preventing arterial thromboembolism, such as in AF, remains hypothetical, as clinical efficacy in reducing thrombotic events has yet to be demonstrated. The PACIFIC-AF trial (NCT04218266) [63], a phase II, including 755 patients with AF meanly aged 73.7 years, 59% men, reported a lower incidence of bleeding with 20 mg or 50 mg of asundexian compared to placebo. Conversely, findings from the larger OCEANIC-AF trial (NCT05643573) [62], including 14,810 patients, meanly aged 73.77 ± 7.7 years, 64,8% of men, revealed an increased risk of stroke or systemic embolism with once-daily asundexian 50 mg relative to apixaban (HR, 3.79; 95% CI, 2.46 to 5.83). In contrast, the LIBREXIA-AF trial (NCT05757869) [64], a global phase III, randomized, double-blind trial with an enrollment target of 15,500 participants with AF, employs milvexian at a twice-daily dosing regimen, using a total daily dose four times higher than that of asundexian in OCEANIC-AF. The aim of the trial is to evaluate the non-inferiority of milvexian compared to apixaban.

The phase II AZALEA-TIMI 71 (NCT04755283) trial [66] was terminated early due to a significantly lower incidence of major and clinically relevant non-major bleeding in patients receiving monthly subcutaneous abelacimab (90 mg or 150 mg) compared with administration of daily rivaroxaban (20 mg). Abelacimab doses achieved sustained suppression of free FXI levels, exceeding a 97% reduction from baseline at 3 months. The primary bleeding endpoint was reduced by 77% in the 90 mg group and by 67% in the 150 mg group, primarily with reductions in major, clinically relevant non-major, and gastrointestinal bleeding events. However, this trial was not powered to evaluate thrombotic efficacy, leaving uncertainty regarding the antithrombotic protection provided by the tested doses. The ongoing phase III LILAC-TIMI 76 trial (NCT05712200) [71] is investigating abelacimab 150 mg monthly versus placebo in approximately 1900 patients with AF who are ineligible for standard anticoagulation. This trial is designed to assess the composite outcome of ischemic stroke or systemic embolism and will monitor bleeding events classified as Bleeding Academic Research Consortium (BARC) type 3c or 5. Table 3 summarizes trials about the role of FXI inhibitors in AF.

Given the heightened thrombotic and bleeding risks in oncology patients with AF, the management of anticoagulation in this population remains particularly challenging. The inclusion of patients with active cancer in the ongoing LILAC-TIMI 76 trial [71] represents a pivotal step toward addressing this unmet clinical need. The results of this study are highly anticipated, as they may offer new insights into the safety and efficacy of FXI inhibition in a high-risk subgroup that has been historically excluded from most anticoagulation trials. If successful, this strategy may pave the way for a paradigm shift in the prevention of cardioembolic events in vulnerable cancer patients with atrial fibrillation.

## 5. Conclusions

The relationship between AF and cancer is complex and bidirectional, influenced by shared risk factors such as chronic inflammation and direct effects of cancer or its treatments. Chronic inflammation, particularly involving the NLRP3 inflammasome, appears to be a central pathophysiological link between the two conditions. Managing AF in cancer patients presents significant challenges due to a heightened risk of both thromboembolism and bleeding, as well as complex drug–drug interactions between antineoplastic agents and anticoagulants. Existing scoring systems like CHA_2_DS_2_-VASc and HAS-BLED have limitations in this specific patient population, and there is a lack of robust evidence from randomized controlled trials to guide optimal management. NOACs have shown promise compared to warfarin in terms of reduced bleeding risks and comparable ischemic stroke rates. Future perspectives in managing AF in cancer patients involve investigating novel anticoagulant strategies, such as FXI inhibitors. A multidisciplinary approach, incorporating the “TBIP” (thromboembolic, bleeding, interactions, patient preferences) strategy, is crucial for making informed treatment decisions in cancer patients with AF.

## Figures and Tables

**Table 1:** Cancer drug classes and proposed mechanisms contributing to atrial fibrillation development.

Drug Class	Proposed Mechanisms
Anthracyclines [22]	Oxidative stress-induced cardiomyocyte damage, ion channel dysfunction, myocarditis and cardiac remodeling, autonomic dysfunction
Tyrosine Kinase Inhibitors [23]	Off-target inhibition of C-terminal Src kinase, structural remodeling and myocardial fibrosis in the atrium, inflammation
Alkylating Agents [24]	Elevated inflammatory markers, alteration of intracellular calcium activity, aggravation of pre-existing pro-arrhythmic conditions
Anti-metabolites [25]	Endothelial dysfunction and vasospasm, oxidative stress, direct myocardial toxicity, electrophysiologic change
HER-2 Antagonists [26]	Disruption of HER2 signaling pathways, structural changes in the heart, cardiac inflammation, and fibrosis
CDK4/6 Inhibitors [27]	Alteration of potassium and sodium channel activity, vascular inflammation
BRAF/MEK Inhibitors [28]	Structural and electrical remodeling in the heart
Immune Checkpoint Inhibitors [29]	Myocarditis, cardiac inflammation leading to arrhythmias, variable incidence based on specific drug and combination therapy
CAR-T-Cell Therapies [30]	Cytokine release syndrome, elevated inflammatory markers
Lenalidomide [31]	Exact mechanism for AF is unknown

**Table 2:** Factor XI inhibitors, the mechanism of action, and the clinical phase of experimentation.

Molecule	Mechanism of Action	The Phase of Clinical Trial
Asundexian	bind to the active site of FXIa	OCEANIC-AF (NCT05643573) phase 3 [62] PACIFIC-AF (NCT04218266) [63]
Milvexian	bind to the active site of FXIa	LIBREXIA-AF trial (NCT05757869) phase 3 [64]
Xisomab	antibodies directed against FXI and FXII	(NCT04465760) Phase 2 [65]
Abelacimab	antibodies directed against FXI	AZALEA-TIMI 71 (NCT04755283) phase 2b [66] ASTER (NCT05171049) phase 3 [67] MAGNOLIA (NCT05171075) phase 3 [68]
Osocimab	antibodies directed against FXI	Phase 2b (NCT04523220) [69]
Fesomersen	antisense oligonucleotides	Phase 2b RE-THINC ESRD (NCT04534114) [70]

FXI: factor XI; FXII: factor XII; RE-THINC ESRD: factor XI LICA to reduce thrombotic events in end-stage renal disease patients on Hemodialysis.

**Table 3:** Main trials about the role of factor XI inhibitors and atrial fibrillation.

Trial	Sample Size (N)	Drugs	Primary Outcome	Results
PACIFIC-AF [63] (NCT04218266)	755	Asundexian	The composite of major or clinically relevant non-major bleeding.	Rate of incidence for the primary endpoint were 0.50 (90% CI 0.14–1.68) for asundexian 20 mg, 0.16 (0.01–0.99) for asundexian 50 mg, and 0.33 (0.09–0.97) for pooled asundexian versus apixaban.
OCEANIC-AF [62] (NCT05643573)	14,810	Asundexian	Superiority of asundexian versus to apixaban to major bleeding events.	The trial was stopped prematurely. During trial, Asundexian at a dose of 50 mg once daily was associated with a higher risk of stroke or systemic embolism when compared than apixaban (hazard ratio, 3.79; 95% CI, 2.46 to 5.83).
LIBREXIA-AF [64] (NCT05757869)	15,500	Milvexian	Non-inferiotity of milvexian versus apixaban for the prevention of stroke and systemic embolism.	Ongoing
AZALEA-TIMI 71 [66] (NCT04755283)	1287	Abelacimab	Major or clinically relevant non-major bleeding.	The trial was stopped early due to a greater-than-anticipated reduction in bleeding events with abelacimab.
LILAC-TIMI 76 [71] (NCT05712200)	1900	Abelacimab	The composite outcome of ischemic stroke or systemic embolism.	Ongoing

AF: atrial fibrillation; CI: confidence interval; mg: milligram.

## Abbreviations

The following abbreviations are used in this manuscript:

AF	Atrial fibrillation
ATE	Arterial thromboembolism
BARC	Bleeding Academic Research Consortium
Cardio-CHUVI-AF	Retrospective Observational Registry of Patients With Atrial Fibrillation From Vigo’s Health Area
CAR-T	Chimeric Antigen Receptor T-cell therapy
CDK4/6	Cyclin-dependent kinase 4 and 6
CHA_2_DS_2_-VA	Congestion, Hypertension, Age ≥ 75, Diabetes mellitus, Vascular disease, Age between 65–74
CI	Confidence interval
CXCR-4	C-X-C chemokine receptor type 4
DAMP	Damage-associated molecular patterns
DDIs	Drug–drug interactions
ECG	Electrocardiogram
ESC	European Society of Cardiology
HAS-BLED	Hypertension, Abnormal liver and renal function, Stroke, Bleeding, Labile international normalized ratio, Elderly, Drugs
HER-2	Human Epidermal Growth Factor Receptor 2
HR	Hazard ratio
MACEs	Major adverse cardiac events
MEK	Mitogen-activated extracellular signal-regulated kinase
NOACs	New oral anticoagulants
P-gp	P-glycoprotein
RR	Relative risk
SGLT2	Sodium–glucose cotransporter 2
STE	Systemic thromboembolism
VKAs	Vitamin K antagonists
VTE	Venous Thromboembolism

## References

[1] Mauriello A., Correra A., Molinari R., Del Vecchio G.E., Tessitore V., D’Andrea A., Russo V. (2024). Mitochondrial Dysfunction in Atrial Fibrillation: The Need for a Strong Pharmacological Approach. Biomedicines.

[2] Van Gelder I.C., Rienstra M., Bunting K.V., Casado-Arroyo R., Caso V., Crijns H.J.G.M., De Potter T.J.R., Dwight J., Guasti L., Hanke T. (2024). 2024 ESC Guidelines for the Management of Atrial Fibrillation Developed in Collaboration with the European Association for Cardio-Thoracic Surgery (EACTS). Eur. Heart J..

[3] Bray F., Laversanne M., Sung H., Ferlay J., Siegel R.L., Soerjomataram I., Jemal A. (2024). Global Cancer Statistics 2022: GLOBOCAN Estimates of Incidence and Mortality Worldwide for 36 Cancers in 185 Countries. CA Cancer J. Clin..

[4] Madnick D.L., Fradley M.G. (2022). Atrial Fibrillation and Cancer Patients: Mechanisms and Management. Curr. Cardiol. Rep..

[5] Benjamin E.J. (1994). Independent Risk Factors for Atrial Fibrillation in a Population-Based Cohort. JAMA.

[6] Mauriello A., Ascrizzi A., Roma A.S., Molinari R., Caturano A., Imbalzano E., D’Andrea A., Russo V. (2024). Effects of Heart Failure Therapies on Atrial Fibrillation: Biological and Clinical Perspectives. Antioxidants.

[7] Lateef N., Kapoor V., Ahsan M.J., Latif A., Ahmed U., Mirza M., Anwar F., Holmberg M. (2020). Atrial Fibrillation and Cancer; Understanding the Mysterious Relationship through a Systematic Review. J. Community Hosp. Intern. Med. Perspect..

[8] Conen D., Wong J.A., Sandhu R.K., Cook N.R., Lee I.-M., Buring J.E., Albert C.M. (2016). Risk of Malignant Cancer Among Women with New-Onset Atrial Fibrillation. JAMA Cardiol..

[9] Jakobsen C.B., Lamberts M., Carlson N., Lock-Hansen M., Torp-Pedersen C., Gislason G.H., Schou M. (2019). Incidence of Atrial Fibrillation in Different Major Cancer Subtypes: A Nationwide Population-Based 12 Year Follow up Study. BMC Cancer.

[10] Farmakis D., Parissis J., Filippatos G. (2014). Insights Into Onco-Cardiology. J. Am. Coll. Cardiol..

[11] Mauriello A., Correra A., Maratea A.C., Caturano A., Liccardo B., Perrone M.A., Giordano A., Nigro G., D’Andrea A., Russo V. (2025). Serum Lipids, Inflammation, and the Risk of Atrial Fibrillation: Pathophysiological Links and Clinical Evidence. J. Clin. Med..

[12] Mauriello A., Correra A., Ascrizzi A., Del Vecchio G.E., Benfari G., Ilardi F., Lisi M., Malagoli A., Mandoli G.E., Pastore M.C. (2024). Relationship Between Left Atrial Strain and Atrial Fibrillation: The Role of Stress Echocardiography. Diagnostics.

[13] Nagai N., Kudo Y., Aki D., Nakagawa H., Taniguchi K. (2021). Immunomodulation by Inflammation during Liver and Gastrointestinal Tumorigenesis and Aging. Int. J. Mol. Sci..

[14] Franceschi C., Garagnani P., Parini P., Giuliani C., Santoro A. (2018). Inflammaging: A New Immune–Metabolic Viewpoint for Age-Related Diseases. Nat. Rev. Endocrinol..

[15] Rositch A.F. (2020). Global Burden of Cancer Attributable to Infections: The Critical Role of Implementation Science. Lancet Glob. Health.

[16] Ullman T.A., Itzkowitz S.H. (2011). Intestinal Inflammation and Cancer. Gastroenterology.

[17] Takahashi H., Ogata H., Nishigaki R., Broide D.H., Karin M. (2010). Tobacco Smoke Promotes Lung Tumorigenesis by Triggering IKKβ- and JNK1-Dependent Inflammation. Cancer Cell.

[18] Quail D.F., Dannenberg A.J. (2019). The Obese Adipose Tissue Microenvironment in Cancer Development and Progression. Nat. Rev. Endocrinol..

[19] Dukić M., Radonjić T., Jovanović I., Zdravković M., Todorović Z., Kraišnik N., Aranđelović B., Mandić O., Popadić V., Nikolić N. (2023). Alcohol, Inflammation, and Microbiota in Alcoholic Liver Disease. Int. J. Mol. Sci..

[20] Gartung A., Yang J., Sukhatme V.P., Bielenberg D.R., Fernandes D., Chang J., Schmidt B.A., Hwang S.H., Zurakowski D., Huang S. (2019). Suppression of Chemotherapy-Induced Cytokine/Lipid Mediator Surge and Ovarian Cancer by a Dual COX-2/SEH Inhibitor. Proc. Natl. Acad. Sci. USA.

[21] Roca H., Jones J.D., Purica M.C., Weidner S., Koh A.J., Kuo R., Wilkinson J.E., Wang Y., Daignault-Newton S., Pienta K.J. (2017). Apoptosis-Induced CXCL5 Accelerates Inflammation and Growth of Prostate Tumor Metastases in Bone. J. Clin. Investig..

[22] Ran T., Chen J., She Q., Mu Y., Zhang M., Mao M., Zuo Z., Li J. (2024). Anthracycline-Induced Arrhythmias in Breast Cancer Therapy: A Meta-Analysis of Single-Arm Trials. PLoS ONE.

[23] Quartermaine C., Ghazi S.M., Yasin A., Awan F.T., Fradley M., Wiczer T., Kalathoor S., Ferdousi M., Krishan S., Habib A. (2023). Cardiovascular Toxicities of BTK Inhibitors in Chronic Lymphocytic Leukemia. JACC CardioOncol..

[24] Albini A., Pennesi G., Donatelli F., Cammarota R., De Flora S., Noonan D.M. (2010). Cardiotoxicity of Anticancer Drugs: The Need for Cardio-Oncology and Cardio-Oncological Prevention. JNCI J. Natl. Cancer Inst..

[25] Dyhl-Polk A., Schou M., Vistisen K.K., Sillesen A.-S., Serup-Hansen E., Faber J., Klausen T.W., Bojesen S.E., Vaage-Nilsen M., Nielsen D.L. (2021). Myocardial Ischemia Induced by 5-Fluorouracil: A Prospective Electrocardiographic and Cardiac Biomarker Study. Oncologist.

[26] Wu W.-C., Huang C.-C., Tsai Y.-F., Lin Y.-S., Feng C.-J., Chen Y.-J., Lai J.-I., Chao T.-C., Liu C.-Y., Tseng L.-M. (2023). The Association of Trastuzumab with Atrial Fibrillation and Heart Failure in Breast Cancer Patients in Routine Clinical Practice: A Population-Based Propensity Score Matching and Competing Risk Model Analysis. Breast Cancer Res. Treat..

[27] Papageorgiou C., Zagouri F., Tampakis K., Georgakopoulou R., Manios E., Kafouris P., Benetos G., Koutagiar I., Anagnostopoulos C., Dimopoulos M.A. (2021). Vascular Inflammation and Cardiovascular Burden in Metastatic Breast Cancer Female Patients Receiving Hormonal Treatment and CDK 4/6 Inhibitors or Everolimus. Front. Cardiovasc. Med..

[28] Glen C., Tan Y.Y., Waterston A., Evans T.R.J., Jones R.J., Petrie M.C., Lang N.N. (2022). Mechanistic and Clinical Overview Cardiovascular Toxicity of BRAF and MEK Inhibitors. JACC CardioOncol.

[29] Zamami Y., Niimura T., Okada N., Koyama T., Fukushima K., Izawa-Ishizawa Y., Ishizawa K. (2019). Factors Associated with Immune Checkpoint Inhibitor–Related Myocarditis. JAMA Oncol..

[30] Shouval R., Goldman A., Flynn J.R., El-Moghraby A., Rehman M., Devlin S.M., Corona M., Landego I., Lin R.J., Scordo M. (2024). Atrial Arrhythmias Following CAR-chimeric Antigen Receptor T-cell Therapy: Incidence, Risk Factors and Biomarker Profile. Br. J. Haematol..

[31] Ahmad J., Thurlapati A., Thotamgari S., Grewal U.S., Sheth A.R., Gupta D., Beedupalli K., Dominic P. (2022). Anti-Cancer Drugs Associated Atrial Fibrillation—An Analysis of Real-World Pharmacovigilance Data. Front. Cardiovasc. Med..

[32] Martinon F., Burns K., Tschopp J. (2002). The Inflammasome. Mol. Cell.

[33] Scott L., Li N., Dobrev D. (2019). Role of Inflammatory Signaling in Atrial Fibrillation. Int. J. Cardiol..

[34] He Y., Hara H., Núñez G. (2016). Mechanism and Regulation of NLRP3 Inflammasome Activation. Trends Biochem. Sci..

[35] Farmakis D. (2021). Anticoagulation for Atrial Fibrillation in Active Cancer: What the Cardiologists Think. Eur. J. Prev. Cardiol..

[36] López-Fernández T. (2023). CHA2DS2-VASc Score in Cardio-Oncology. JACC CardioOncol.

[37] D’Souza M., Carlson N., Fosbøl E., Lamberts M., Smedegaard L., Nielsen D., Torp-Pedersen C., Gislason G., Schou M. (2018). CHA_2_ DS_2_-VASc Score and Risk of Thromboembolism and Bleeding in Patients with Atrial Fibrillation and Recent Cancer. Eur. J. Prev. Cardiol..

[38] Leader A., Cohen N.M., Afek S., Jaschek R., Frajman A., Ben Zadok O.I., Raanani P., Lishner M., Spectre G. (2023). Arterial Thromboembolism in Patients with AF and CHA_2_DS_2_-VASc Score 0–2 with and without Cancer. JACC CardioOncol.

[39] Raposeiras-Roubin S., Abu-Assi E., Marchán A., Fernández-Sanz T., Barreiro-Pardal C., Pousa I.M., Erquicia P.D., Ledo-Piñeiro A., González-Bermúdez I., Viu M.M. (2022). Validation of Embolic and Bleeding Risk Scores in Patients with Atrial Fibrillation and Cancer. Am. J. Cardiol..

[40] Jalaber E., Orvain C., Papadopoulou V., Genthon A., Daguerre V., Barrière S., Teste A., Tavernier E., Daguenet E., Chalayer E. (2025). Management of Thrombocytopenia and Anticoagulant Therapy in Patients with Hematological Malignancy on Chemotherapy: A Binational Prospective Study (TAT Study). J. Thromb. Thrombolysis.

[41] Fradley M.G., Beckie T.M., Brown S.A., Cheng R.K., Dent S.F., Nohria A., Patton K.K., Singh J.P., Olshansky B. (2021). Recognition, Prevention, and Management of Arrhythmias and Autonomic Disorders in Cardio-Oncology: A Scientific Statement From the American Heart Association. Circulation.

[42] Beavers C.J., Rodgers J.E., Bagnola A.J., Beckie T.M., Campia U., Di Palo K.E., Okwuosa T.M., Przespolewski E.R., Dent S. (2022). Cardio-Oncology Drug Interactions: A Scientific Statement From the American Heart Association. Circulation.

[43] Steffel J., Collins R., Antz M., Cornu P., Desteghe L., Haeusler K.G., Oldgren J., Reinecke H., Roldan-Schilling V., Rowell N. (2021). 2021 European Heart Rhythm Association Practical Guide on the Use of Non-Vitamin K Antagonist Oral Anticoagulants in Patients with Atrial Fibrillation. Europace.

[44] Wiggins B.S., Dixon D.L., Neyens R.R., Page R.L., Gluckman T.J. (2020). Select Drug-Drug Interactions with Direct Oral Anticoagulants. J. Am. Coll. Cardiol..

[45] Gnoth M.J., Buetehorn U., Muenster U., Schwarz T., Sandmann S. (2011). In Vitro and In Vivo P-Glycoprotein Transport Characteristics of Rivaroxaban. J. Pharmacol. Exp. Ther..

[46] Mueck W., Kubitza D., Becka M. (2013). Co-administration of Rivaroxaban with Drugs That Share Its Elimination Pathways: Pharmacokinetic Effects in Healthy Subjects. Br. J. Clin. Pharmacol..

[47] Mauriello A., Ascrizzi A., Molinari R., Falco L., Caturano A., D’Andrea A., Russo V. (2023). Pharmacogenomics of Cardiovascular Drugs for Atherothrombotic, Thromboembolic and Atherosclerotic Risk. Genes.

[48] Shah S., Norby F.L., Datta Y.H., Lutsey P.L., MacLehose R.F., Chen L.Y., Alonso A. (2018). Comparative Effectiveness of Direct Oral Anticoagulants and Warfarin in Patients with Cancer and Atrial Fibrillation. Blood Adv..

[49] Lee Y.-J., Park J., Uhm J.-S., Kim J.-Y., Pak H.-N., Lee M.-H., Sung J.-H., Joung B. (2016). Bleeding Risk and Major Adverse Events in Patients with Cancer on Oral Anticoagulation Therapy. Int. J. Cardiol..

[50] Deitelzweig S., Keshishian A.V., Zhang Y., Kang A., Dhamane A.D., Luo X., Klem C., Ferri M., Jiang J., Yuce H. (2021). Effectiveness and Safety of Oral Anticoagulants Among Nonvalvular Atrial Fibrillation Patients With Active Cancer. JACC CardioOncol.

[51] Mariani M.V., Magnocavallo M., Straito M., Piro A., Severino P., Iannucci G., Chimenti C., Mancone M., Rocca D.G.D., Forleo G.B. (2021). Direct Oral Anticoagulants versus Vitamin K Antagonists in Patients with Atrial Fibrillation and Cancer a Meta-Analysis. J. Thromb. Thrombolysis.

[52] Lee A.Y.Y., Levine M.N., Baker R.I., Bowden C., Kakkar A.K., Prins M., Rickles F.R., Julian J.A., Haley S., Kovacs M.J. (2003). Low-Molecular-Weight Heparin versus a Coumarin for the Prevention of Recurrent Venous Thromboembolism in Patients with Cancer. N. Engl. J. Med..

[53] Granger C.B., Alexander J.H., McMurray J.J.V., Lopes R.D., Hylek E.M., Hanna M., Al-Khalidi H.R., Ansell J., Atar D., Avezum A. (2011). Apixaban versus Warfarin in Patients with Atrial Fibrillation. N. Engl. J. Med..

[54] Patel M.R., Mahaffey K.W., Garg J., Pan G., Singer D.E., Hacke W., Breithardt G., Halperin J.L., Hankey G.J., Piccini J.P. (2011). Rivaroxaban versus Warfarin in Nonvalvular Atrial Fibrillation. N. Engl. J. Med..

[55] Connolly S.J., Ezekowitz M.D., Yusuf S., Eikelboom J., Oldgren J., Parekh A., Pogue J., Reilly P.A., Themeles E., Varrone J. (2009). Dabigatran versus Warfarin in Patients with Atrial Fibrillation. N. Engl. J. Med..

[56] Giugliano R.P., Ruff C.T., Braunwald E., Murphy S.A., Wiviott S.D., Halperin J.L., Waldo A.L., Ezekowitz M.D., Weitz J.I., Špinar J. (2013). Edoxaban versus Warfarin in Patients with Atrial Fibrillation. N. Engl. J. Med..

[57] Pastori D., Marang A., Bisson A., Menichelli D., Herbert J., Lip G.Y.H., Fauchier L. (2021). Thromboembolism, Mortality, and Bleeding in 2,435,541 Atrial Fibrillation Patients with and without Cancer: A Nationwide Cohort Study. Cancer.

[58] Hu Y., Liu C., Chang P.M., Tsao H., Lin Y., Chang S., Lo L., Tuan T., Li C., Chao T. (2013). Incident Thromboembolism and Heart Failure Associated with New-Onset Atrial Fibrillation in Cancer Patients. Int. J. Cardiol..

[59] Mohsin S., Hasan M., Sheikh Z.M., Mustafa F., Tegeltija V., Kumar S., Kumar J. (2024). Efficacy of SGLT2 Inhibitors for Anthracycline-Induced Cardiotoxicity: A Meta-Analysis in Cancer Patients. Future Cardiol..

[60] Kommu S. (2024). The Role of SGLT2 Inhibitors on Heart Failure Outcomes in Nondiabetic Patients: A Systematic Review and Meta-Analysis of Randomized Controlled Trials. J. Cardiovasc. Pharmacol..

[61] Quagliariello V., Di Mauro A., Ferrara G., Bruzzese F., Palma G., Luciano A., Canale M.L., Bisceglia I., Iovine M., Cadeddu Dessalvi C. (2025). Cardio–Renal and Systemic Effects of SGLT2i Dapagliflozin on Short-Term Anthracycline and HER-2-Blocking Agent Therapy-Induced Cardiotoxicity. Antioxidants.

[62] Piccini J.P., Patel M.R., Steffel J., Ferdinand K., Van Gelder I.C., Russo A.M., Ma C.S., Goodman S.G., Oldgren J., Hammett C. (2024). Asundexian versus Apixaban in Patients with Atrial Fibrillation. N. Engl. J. Med..

[63] Shoamanesh A., Mundl H., Smith E.E., Masjuan J., Milanov I., Hirano T., Agafina A., Campbell B., Caso V., Mas J.L. (2022). Factor XIa Inhibition with Asundexian after Acute Non-Cardioembolic Ischaemic Stroke (PACIFIC-Stroke): An International, Randomised, Double-Blind, Placebo-Controlled, Phase 2b Trial. Lancet.

[64] Jain S.S., Mahaffey K.W., Pieper K.S., Shimizu W., Potpara T., Ruff C.T., Kamel H., Lewis B.S., Cornel J.H., Kowey P.R. (2024). Milvexian vs. Apixaban for Stroke Prevention in Atrial Fibrillation: The LIBREXIA Atrial Fibrillation Trial Rationale and Design. Am. Heart J..

[65] Pfeffer M.A., Kohs T.C.L., Vu H.H., Jordan K.R., Wang J.S.H., Lorentz C.U., Tucker E.I., Puy C., Olson S.R., DeLoughery T.G. (2024). Factor XI Inhibition for the Prevention of Catheter-Associated Thrombosis in Patients with Cancer Undergoing Central Line Placement: A Phase 2 Clinical Trial. Arter. Thromb. Vasc. Biol..

[66] Ruff C.T., Patel S.M., Giugliano R.P., Morrow D.A., Hug B., Kuder J.F., Goodrich E.L., Chen S.A., Goodman S.G., Joung B. (2025). Abelacimab versus Rivaroxaban in Patients with Atrial Fibrillation. N. Engl. J..

[67] ClinicalTrials.gov (2024). A Study Comparing Abelacimab to Apixaban in the Treatment of Cancer-Associated VTE (ASTER). https://www.clinicaltrials.gov/study/NCT05171049.

[68] ClinicalTrials.gov (2024). A Study Comparing Abelacimab to Dalteparin in the Treatment of Gastrointestinal/Genitourinary Cancer and Associated VTE (MAGNOLIA). https://clinicaltrials.gov/study/NCT05171075.

[69] Weitz J.I., Tankó L.B., Floege J., Fox K.A.A., Bhatt D.L., Thadhani R., Hung J., Pap Á.F., Kubitza D., Winkelmayer W.C. (2024). Anticoagulation with Osocimab in Patients with Kidney Failure Undergoing Hemodialysis: A Randomized Phase 2 Trial. Nat. Med..

[70] Winkelmayer W.C., Lensing A.W.A., Thadhani R.I., Mahaffey K.W., Walsh M., Pap Á.F., Willmann S., Thelen K., Hodge S., Solms A. (2024). A Phase II Randomized Controlled Trial Evaluated Antithrombotic Treatment with Fesomersen in Patients with Kidney Failure on Hemodialysis. Kidney Int..

[71] ClinicalTrials.gov Study to EvaLuate the EffIcacy and Safety of AbeLacimab in High-Risk Patients with Atrial Fibrillation Who Have Been Deemed Unsuitable for Oral AntiCoagulation (LILAC-TIMI 76) (LILAC-TIMI 76). https://clinicaltrials.gov/study/NCT05712200.

